# Injury to the posterior malleolus in Maisonneuve fractures

**DOI:** 10.1007/s00068-023-02394-7

**Published:** 2023-12-02

**Authors:** Michal Tuček, Jan Bartoníček, Petr Fojtík, Konrad Kamin, Stefan Rammelt

**Affiliations:** 1 Department of Orthopaedics, First Faculty of Medicine, Charles University and Military University Hospital Prague, U Vojenské Nemocnice 1200, Prague 6, 169 02 Czech Republic; 2 University Center for Orthopaedics, Trauma and Plastic Surgery, University Hospital Carl Gustav Carus at TU Dresden, Fetscherstrasse 74, 01307 Dresden, Germany

**Keywords:** Maisonneuve fracture, Posterior malleolus, Fibular notch, Tillaux–Chaput tubercle

## Abstract

**Purpose:**

The aim of this study was to describe the incidence and a complex pathoanatomy of posterior malleolus fractures in a Maisonneuve fracture.

**Methods:**

The study included 100 prospectively collected patients with a complete clinical and radiological documentation of an ankle fracture or fracture-dislocation including a fracture of the proximal quarter of the fibula.

**Results:**

A posterior malleolus fracture was identified in 74 patients, and in 27% of these cases it carried more than one quarter of the fibular notch. Displacement of the posterior fragment by more than 2 mm was shown by scans in 72% of cases. Small intercalary fragments were identified in 43% of cases. Fractures of the Tillaux–Chaput tubercle were identified in 20 patients.

**Conclusion:**

Our study has proved a high rate of posterior malleolus fractures associated with a Maisonneuve fracture, and documented their considerable variability in terms of involvement of the fibular notch, tibiotalar contact area, direction of displacement and frequency of intercalary fragments. Of no less importance is a combination of Tillaux–Chaput fractures with a Maisonneuve fracture.

## Introduction

Maisonneuve fracture (MF) is a generally known, although not very frequent type of ankle fracture-dislocation [1–4]. Recent studies have shown that it is an injury with a highly variable pathoanatomy, associated in about 80% of cases with a fracture of the posterior malleolus (PM) [2, 3, 5, 6]. This fact is very important as reduction and fixation of a displaced PM may considerably facilitate anatomical reduction of the distal fibula into the fibular notch (FN). The few existing studies on that topic [2, 5] have mentioned PM injury in MF only briefly, therefore we have decided to focus on this issue on a large patient cohort and in greater detail.

## Materials and methods

Between January 2012 and April 2022 we collected prospectively and evaluated 117 patients with MF treated at our institution. MF was defined as an ankle fracture or fracture-dislocation, including a fracture of the proximal quarter of the fibula. Full radiological documentation, i.e., radiographs and computed tomography (CT) scans, was available for all patients. Excluded from the study group were 17 patients with MF, due to skeletal immaturity, with previous injuries or interventions at the ankle, ankle osteoarthritis or a two-level fibular fracture (“double Maisonneuve fracture”) [7].

The final study group thus comprised 100 patients with the mean age of 51.2 years (range, 26–84). Among these were 67 men with the mean age of 47.8 years (range, 26–78) and 33 women with the mean age of 58.3 years (range, 39–84). The right side was involved in 56 and the left side in 44 cases. Non-operative treatment was employed in 16 patients, and 84 patients were treated operatively, including 23 individuals treated with open reduction and direct internal fixation of a displaced PM from a posterior approach (posterolateral approach in 17 and posteromedial approach in 6 patients).

### Methods

All the patients underwent complete radiological examination, i.e., anteroposterior (ap), mortise and lateral views of the ankle, ap and lateral views of the lower leg and CT scanning, including axial, coronal, sagittal scans and 3D CT reconstructions. Radiological evaluation was performed always by the first two authors, in case of different opinions, the respective case was discussed by all authors in order to reach agreement.

### Assessment

The following parameters were assessed on the basis of radiological documentation.

- PM fracture incidence and type according to the Bartoníček–Rammelt (B–R) classification [8],
- cross-sectional area of the PM fragment;
- involvement of the fibular notch (FN) on CT scans [2, 3, 9];
- PM fragment displacement on axial and sagittal scans, and 3D CT reconstructions (fractures with a displacement of less than 2 mm in all parts of the fragment were classified as nondisplaced);
- incidence, location and displacement of intercalary fragments in individual types of PM fractures [10];
- injuries to the medial structures (MS), i.e. rupture of the deltoid ligament, fracture of the medial malleolus or a combined (osteoligamentous) lesion;
- fracture of the Tillaux–Chaput tubercle (TCT) classified after Rammelt et al. [11].

## Results

### Incidence and type of PM fractures

A PM fracture was identified in 74 patients (74%) with a MF. In 70 cases, it was detected on plain radiographs. In four cases it could be seen only on CT scans, all of those were non-displaced fractures of type 1 (1 case) and type 2 (3 cases) of the B–R classification.

Individual types of the B–R classification [8] were represented in 74 patients as follows: type 1 occurred in 12 cases (16%), type 2 in 44 cases (60%), type 3 in 15 patients (20%) and type 4 in 3 cases (4%).

The exact size of the PM fragment could be assessed on CT scans in 73 of 74 patients, only in 1 case of type 1 the bone was completely crushed. The size of the cross-sectional area of the PM fragment and involvement of the fibular notch by individual types are shown in Table 1.

**Table 1:** Fragment size in individual types of PM fractures according to B–R classification

	CSA	FNI	N FNI ≥ 25%	N FNI ≥ 33%
Type 1	11.2% (8–18%)	–	–	–
Type 2	18.2% (13–30%)	19.3% (9–44%)	8	4
Type 3	26.1% (14–43%)	28.2% (15–45%)	10	4
Type 4	32.7% (24–47%)	38.7% (19–50%)	2	2

*CSA* cross-sectional area; *FNI* fibular notch involvement; *N* number of cases

### Displacement of the fractured PM

**Fig. 1:**
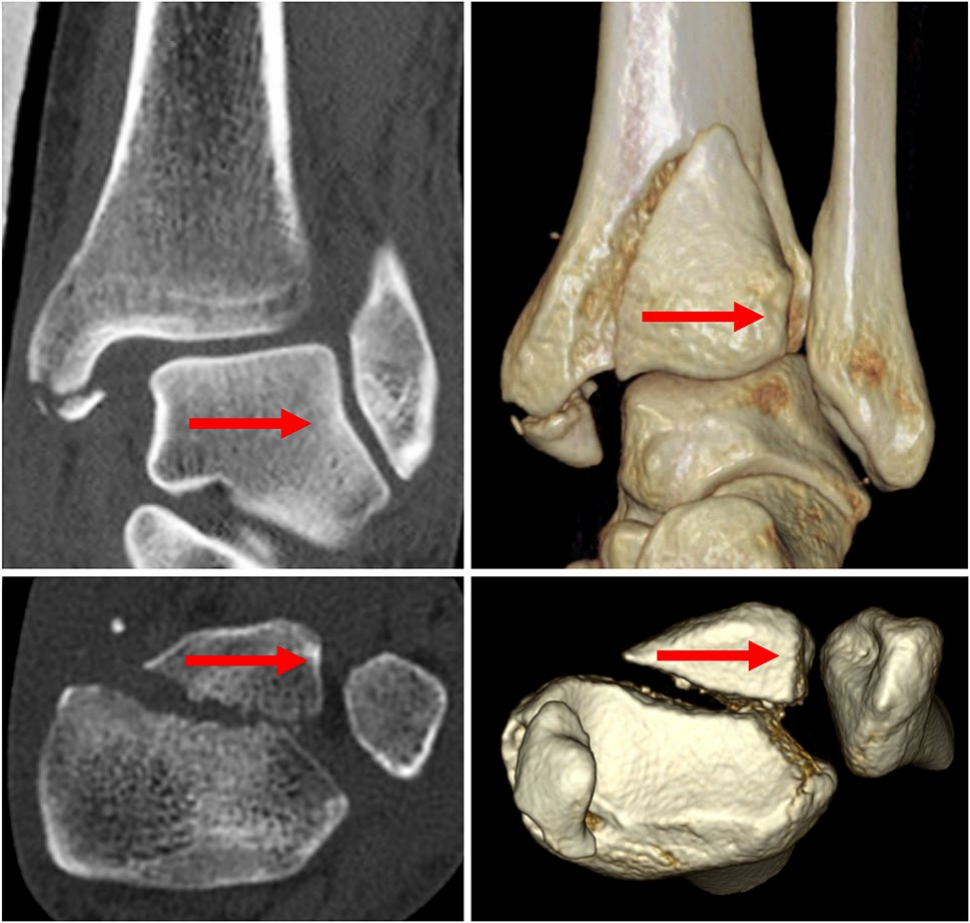
Lateral displacement of PM fragment together with the distal fibula

**Fig. 2:**
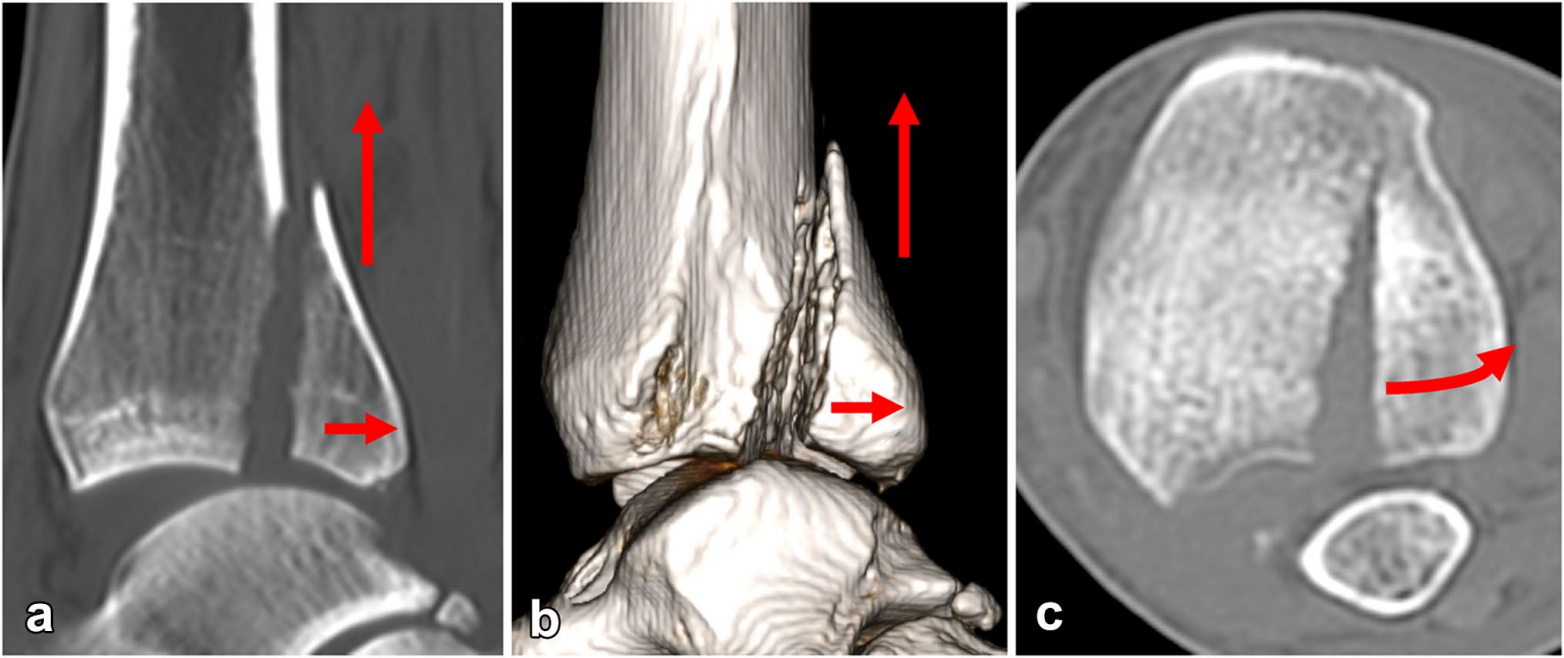
Proximal and posterior displacement of PM (**a, b**) and lateral opening of fracture line with a medial hinge (**c**)

Displacement of the PM fragment by more than 2 mm was recorded on CT scans in 53 cases (72%). We identified three basic directions of displacement: lateral (Fig. 1), proximal and fracture line opening laterally, with a medial hinge (Fig. 2).

In 22 patients, displacement by more than 2 mm was measurable in one of the basic directions only. In 31 cases, the fragment was displaced in 2–3 basic directions. In these cases, the resulting displacement of the PM fragment was always a combination of a shift or rotation in several directions: lateral displacement, proximal displacement, posterior displacement, rotation in the axial plane around a medial hinge.

**Fig. 3:**
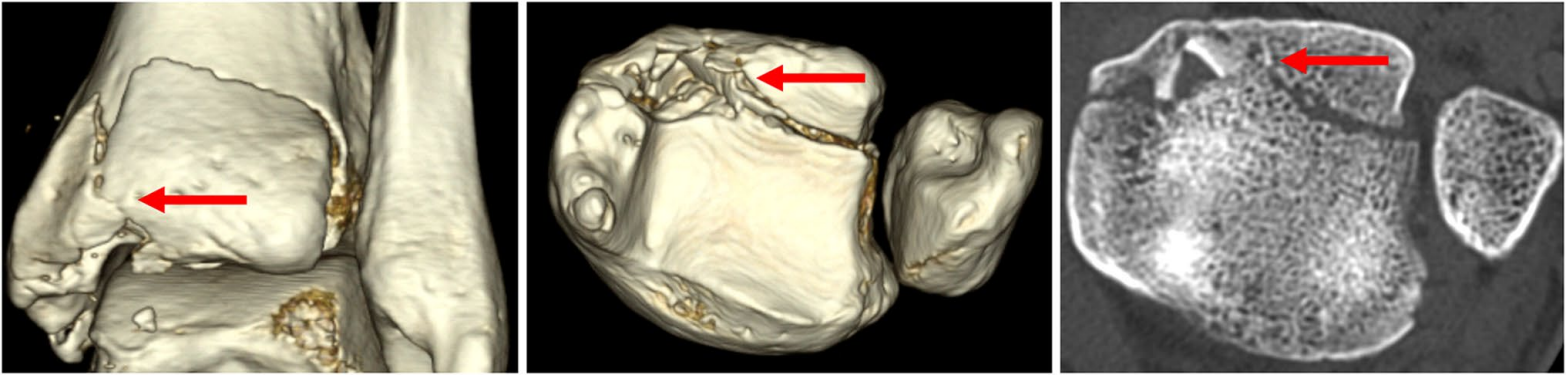
Medial shift of PM fragment

Overall, proximal displacement was detected in 25 cases, lateral displacement in 13 cases, posterior displacement in 27 cases and fracture line opening laterally with a medial hinge in 20 patients. In one patient with a B–R type 3 fracture, the PM fragment was displaced proximally, posteriorly and medially (Fig. 3).

### Involvement of FN

**Fig. 4:**
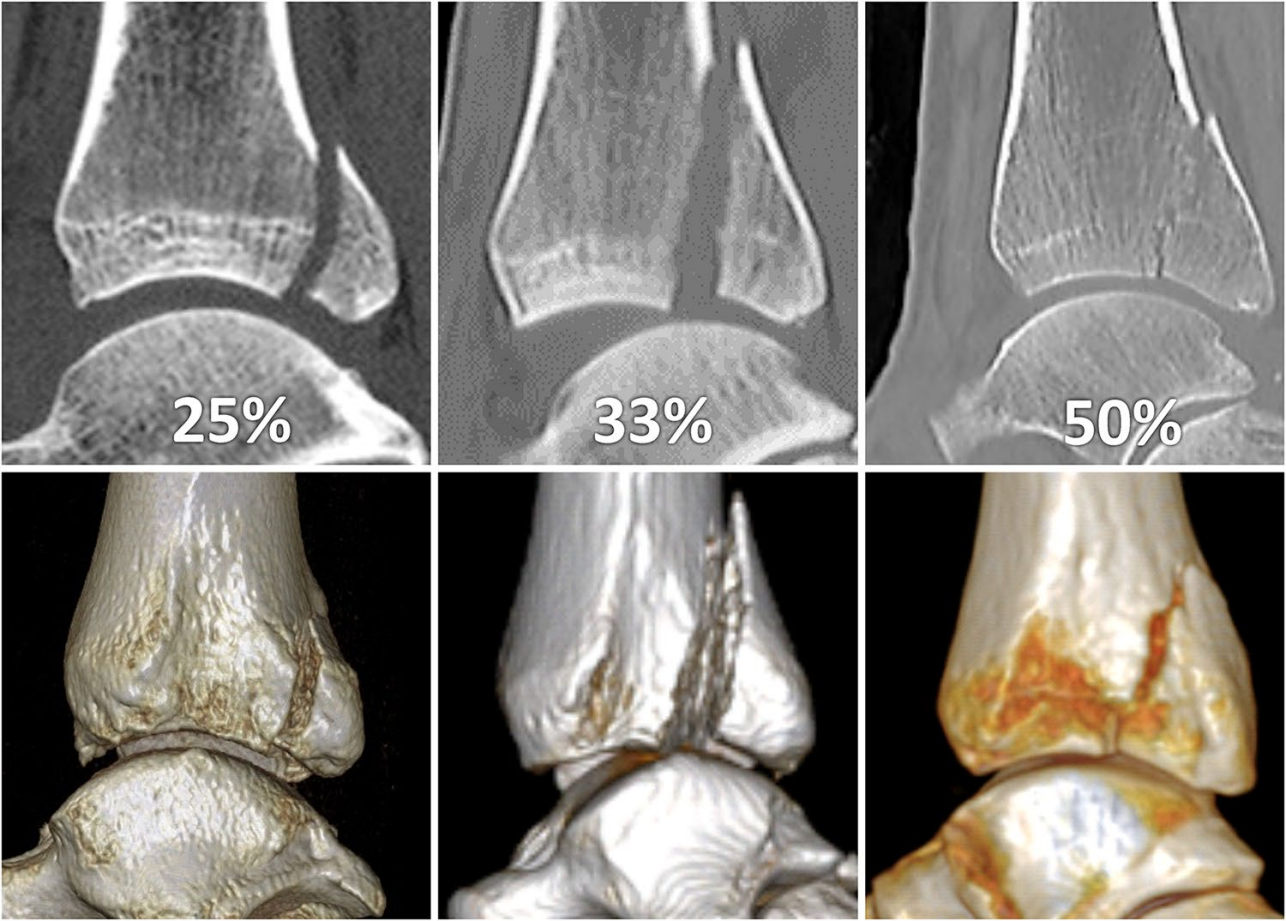
Increasing involvement of fibular notch by fracture of PM on 2D and 3D CT (lateral view with fibula subtraction) reconstructions

The posterior malleolar fragment carried more than one quarter of FN in 20 patients (27% of all PM fractures) (Fig. 4) and more than one third of FN in 10 patients (14% of all PM fractures) (Table 1). Reduction and fixation of a displaced PM via a posterior approach was performed in 23 patients. Of these, a total of 15 patients showed involvement of FN ≥ 25% and eight patients ≤ 25%.

### Incidence of intercalary fragments

**Fig. 5:**
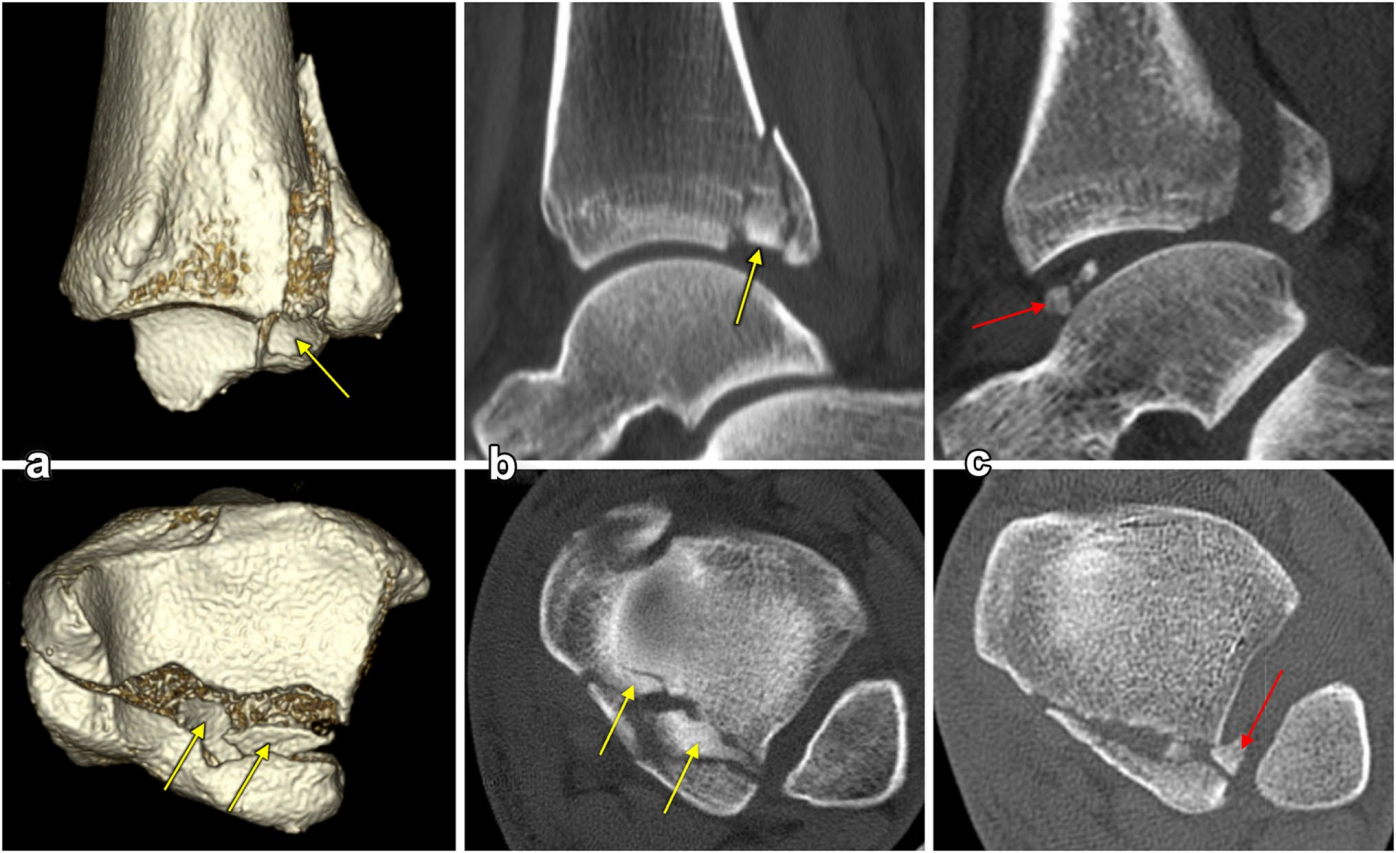
Distributions of intercalary fragments. **a, b** in fracture line of PM (yellow arrows), **c** in joint cavity (red arrows)

Small intercalary fragments (ICF) were identified on CT scans in 32 of 74 cases (43%) of PM fractures. Of these, 24 (75%) were displaced (Fig. 5). Intercalary fragments were most often found in B–R type 2 fractures: 24 of 44 cases (55%), followed by type 3 in 7 of 15 cases (47%) and type 4 in 1 of 3 cases (33%). In 27 of 32 cases (84%), the fragments were located in zone 7 or 8 [10]. In another eight cases small bone fragments were displaced into the fibular notch or tibio-talar articulation (Fig. 5).

### Injuries to medial structures

Injury to the deltoid ligament (DL) or fracture of the medial malleolus (MM) were diagnosed in 82 cases (82%). A DL rupture, defined as a medial clear space of more than 4 mm on ap radiograph or CT coronal scan, or a positive external rotation test at the beginning of operation, was found in 39 patients (39%). A bicollicular MM fracture was recorded in 24 cases (24%). An osteoligamentous lesion of the MS, i.e., fracture of the anterior colliculus and rupture of the tibiotalar part of the DL (as evidenced by medial clear space between the intact posterior colliculus of the MM and talar dome of more than 4 mm) was observed in 19 cases (19%). The types of injuries to MS in individual PM fracture patterns are shown in Table 2.

**Table 2:** Injuries to medial structures in individual types of PM fractures according to the B-R classification

	PM 0	PM 1	PM 2	PM 3	PM 4	Cohort
Intact	12	1	3	1	1	18
DL	7	8	18	4	2	39
BC	4	1	15	4	0	24
Osseo-Lig	3	2	8	6	0	19
Total	26	12	44	15	3	100

*DL* deltoid ligament; *BC* bicollicular fracture of medial malleolus; *Osseo-Lig* osseoligamentous lesion, i.e., fracture of anterior colliculus and rupture of tibiotalar part of deltoid ligament

### Fracture of the Tillaux–Chaput tubercle (anterior malleolus, AM)

This injury was identified in 20 patients (20%). The AM fractures were detected on plain radiographs in three cases (one case of type 1 and two cases of type 2 of the Rammelt classification [11]); 17 cases were revealed only by CT. Overall, 15 cases were classified as Rammelt type 1 and 5 cases as type 2. No Rammelt type 3 fracture (impaction of the lateral plafond) was seen. In 17 cases this injury was associated with a PM fracture, including 13 cases of type 2, 3 cases of type 1 and 1 case of type 4 according to the B–R classification.

## Discussion

In a CT-based analysis of 100 patients with Maisonneuve fractures (MF) we found associated posterior malleolus (PM) fractures in almost three quarters of cases (74%). The incidence of PM fractures in MF has been mentioned in several previous studies with considerable variations. Some of them presented low incidence rates in a range of 35–37% [12–14], while others published substantially higher incidences in the range of 77–83% [2, 5, 15–17]. Only 4 fractures (5%) in our study group were not detected on plain radiographs, all of them being small fragments of type 1 or 2 of the B-R classification. In contrast, He et al. [5] presented 17% of PM fractures seen only on CT scans. Good quality radiographs with exact projections are a prerequisite for detecting PM fractures. These may be difficult to obtain in the setting of an acute fracture with a painful ankle that is immobilized in a splint or cast. The indication to CT scanning should therefore be made generously if a PM fracture is suspected [3].

The share of PM fractures in individual patterns of ankle fracture-dislocations varies considerably. Jedlička et al. [1], in a radiographic study of 232 patients with ankle fracture-dislocations, found a PM fracture in 4% of Weber type A and in 46% of Weber type B and C fractures. Kostlivý et al. [18] identified a PM fracture in 70% of 110 cases of Bosworth fracture-dislocation (BF). These findings point to a higher severity of MF and BF as compared to other types of ankle fracture-dislocations.

Comparison of individual types of PM fractures with previous studies [2, 5] showed certain differences. Bartoníček et al. [6] when analyzing 141 consecutive cases of a PM fractures found a ratio of types 1 and 2 (milder types resulting merely from rotation) to types 3 and 4 (more severe types with a compression component) of 1.6:1, while in the present series of MF it was 3.2:1 (Table 3). Similar to our results, the most frequent PM fragment morphology was type 2 of the B–R classification.

**Table 3:** Comparison of shares of individual types of PM fractures between the series of 141 trimalleolar fractures [8] and the series of 100 Maisonneuve fractures

B-R Type	Bar 2015 141 trimall fxs (%)	Authors 100 MF
Type 1	8	16
Type 2	52	60
Type 3	28	20
Type 4	9	4
Type 5	3	0

Besides the size of PM fragment, in terms of involvement of the articular surface, involvement of FN and the presence and dislocation of ICFs as a criterion for operative treatment has been introduced only recently [3, 10].

**Fig. 6:**
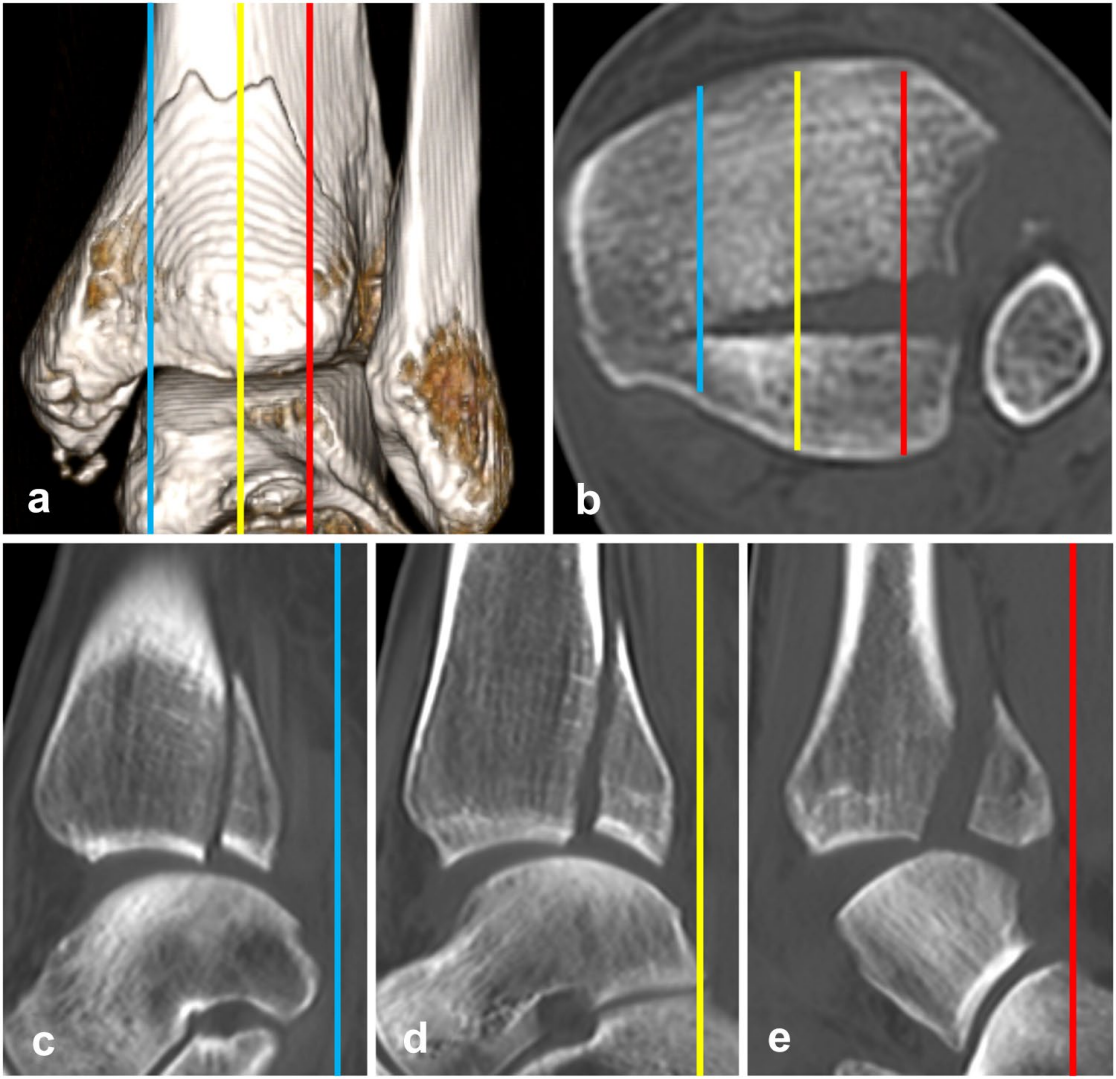
The size of PM fragment and its articular surface depending on location sagittal CT scans. **a** 3D CT posterior view; **b** axial CT scans; **c** medial (blue line) sagittal CT scan; **d** central (yellow line) sagittal scan; **e** lateral (red line) sagittal scan

As the percentage of the articular surface carried by the PM fragment increases from medial to lateral on the sagittal scans, measurements have to be standardized (Fig. 6). The same applies to assessment of FN involvement which is based on axial scans, because the values increase from proximal to distal direction [6, 9]. Based on the anatomical study by Fojtík et al. [19], we measured the values 5 mm proximal to the tibio-talar joint line, where FN is the deepest. The best way to measure the size of PM fragment including involvement of FN are 3D CT reconstruction views of FN with subtraction of the fibula (Fig. 4) and in mortise view with subtraction of the talus (Fig. 1).

In our series, ≥ 33% of FN was involved in 14% of all PM fractures and 25–33% of FN in 27% of all PM fractures. Reduction and fixation of a displaced PM from the posterior approach were performed in 15 patients with ≥ 25% and in eight patients with ≤ 25% of FN involvement.

Displacement of the PM fragment is an indication criterion for direct internal fixation from the posterior approach [3, 4, 6, 20–25]. However, the direction of displacement has not yet been described in the literature. In a majority of cases (58%), fragments showed complex displacement in several directions.

The most important from the clinical viewpoint is, in our view, proximal or lateral displacement. A proximally displaced fragment as seen in 47% of cases in the present study reduces the size of the tibiotalar articular surface. A laterally displaced fragment found in 25% of cases leads to additional malalignment in FN and, as a result, prevents reduction of the distal fibula resulting in an incongruent ankle mortise.

We recorded solid intraarticular ICF in 43% of cases, similarly as Mueller et al. [10] who found ICF in 41% of PM fractures in tri- and quadrimalleolar fractures, most frequently in types 2 and 3 of the B–R classification. These fragments were most often observed in the region of the fracture line, in zones 7 and 8. In another 8 cases we found minor bone chips extruded into FN or the tibio-talar joint space, where, similarly as ICF, they may act as a mechanical obstacle.

The Tillaux–Chaput tubercle which carries the anterior syndesmosis shares many features with the posterior malleolus and may be considered a 4th or anterior malleolus [11]. In the present study, 17 of 100 MF had a concomitant anterior and posterior malleolar fracture which would be considered a quadrimalleolar fracture or pronation external rotation stage 4 according to the Lauge–Hansen classification. Taken together with the more severe types of PM fractures, this shows the relatively high energy producing a MF.

Our results point to a high variability of the shape, size and displacement of PM fragments associated with MF, as well as to their clinical importance from the viewpoint of impairment of FN integrity. Restoration of FN integrity is one of the basic prerequisites of anatomical reduction of the distal fibula into FN. Such reduction is also one of the basic factors influencing clinical and radiological results of MF treatment.

For this reason, all MFs should be examined by CT which will show pathoanatomy of a PM fragment, that is essential for the choice of treatment method, and may also reveal additional injuries not detected by radiographs [3]. Further research evaluating individual MF subtypes is required to establish clear criteria for operative treatment of individual lesions within MF.

One of the benefits of our study is the high number of prospectively included patients and their standardized CT evaluation, while a relative disadvantage may be absence of MRI examination providing important details about injuries to ligamentous structures.

## Conclusion

Our study has proved a high rate of PM fractures (74%) associated with MF, and documented their considerable variability in terms of involvement of FN, tibiotalar contact area and direction of displacement. Most of them were of type 2 of the B–R classification. The rate of more severe PM fractures (types 3 and 4) was twice as high as in a comparable study looking at all types of malleolar fractures. A high incidence and displacement of ICF and AM fractures should be taken into account during preoperative planning.

CT scans in axial, sagittal and coronal planes should be a standard part of radiological examination in MF as they allow, among other things, also to determine the type, size and displacement of the PM fragment, i.e., details essential for planning of the operative treatment.
